# A social-ecological engagement with reef passages in New Caledonia: Connectors between coastal and oceanic spaces and species

**DOI:** 10.1007/s13280-022-01762-8

**Published:** 2022-08-18

**Authors:** Annette Breckwoldt, Yvy Dombal, Catherine Sabinot, Gilbert David, Léa Riera, Sebastian Ferse, Elodie Fache

**Affiliations:** 1 Social-Ecological Systems Analysis, Social Science Department, Leibniz Centre for Tropical Marine Research (ZMT), Fahrenheitstrasse 6, 28359 Bremen, Germany; 2 ESPACE-DEV, IRD, Université de Montpellier, Université des Antilles, Université de Guyane, Université de la Réunion, Université de la Nouvelle-Calédonie, 6B, 161 Pouembout, route de la forêt sèche, BP 440, 98825 Pouembout, New Caledonia France; 3 ESPACE-DEV, Centre IRD de Nouméa, BPA5, 98848 Nouméa Cedex, New Caledonia France; 4 ESPACE-DEV, IRD, Université de Montpellier, Université des Antilles, Université de Guyane, Université de la Réunion, Université de la Nouvelle-Calédonie, 500 rue Jean-François Breton, 34093 Montpellier, France; 5 SENS, IRD, CIRAD, Université Paul Valery Montpellier 3, Université de Montpellier, Site St Charles 2, 71 rue Professeur Henri Serre, 34086 Montpellier, France; 6 Science Management/Social Science Department, Office for Knowledge Exchange (OKE), Leibniz Centre for Tropical Marine Research (ZMT), Fahrenheitstrasse 6, 28359 Bremen, Germany

**Keywords:** Coral reef passages, Divers, Fishers, Marine conservation, New Caledonia, Social and ecological roles

## Abstract

Healthy and protected coral reefs help island systems in the tropics thrive and survive. Reef passages link the open ocean to lagoon and coastal areas in these ecosystems and are home to an exceptionally diverse and abundant marine life, hosting emblematic species and fish spawning aggregations. Their multiple benefits for the islands and their peoples (e.g., for transport, fishing, socio-cultural aspects) remain yet understudied. Drawing from qualitative interviews with fishers, scuba divers, and surfers along the coast of Grande Terre in New Caledonia, this study highlights the multi-faceted importance of these keystone places. It shows that reef passages are locally deemed ‘communication zones’ between coastal and oceanic spaces and species, and have significant un(der)explored ecological and socio-cultural roles. Understanding and protecting these ecological and cultural keystone places will strengthen both the reef ecosystems and the people dependent on them.

**Supplementary Information:**

The online version contains supplementary material available at 10.1007/s13280-022-01762-8.

## Introduction

The United Nations’ Sustainable Development Goal (SDG) 14 – Life below water – aims to support and encourage action to conserve and sustainably use the oceans. This SDG is entangled with the global target to expand conservation areas to 30% of the Earth’s surface by 2030. With this 30% target focusing on “areas particularly important for biodiversity” (CBD 2020), coral reefs, on which coastal people depend to thrive and survive, are high on the agenda.^1^ France, for example, aims to designate 100% of its coral reefs – mainly located in its overseas territories such as New Caledonia, which hosts the second-largest barrier reef in the world (Payri 2019) – as protected areas by 2025, whilst also paying particular attention to the land-sea interface related to reefs for potential “strict protection” (French State 2021:17, 20).

In these biodiversity-rich reef ecosystems, reef passages are openings or channels that connect sheltered lagoon and coastal areas to the open ocean through complex hydrodynamic processes depending on tides, currents, waves, and wind (e.g., Herdman et al. 2017; Sous et al. 2017). These passages and processes are essential to the renewal of water in lagoons, as well as for the transport and redistribution of food items for planktonic and nektonic feeding fishes, and of eggs and developing larvae of spawning reef fishes (Fisher et al. 2018). Many species (e.g., the longnose trevally, *Carangoides chrysophrys*) use reef passages to move between feeding areas, with their predictable movements often being related to both tidal flows and lunar cycles (Hamilton and Walter 1999). Most importantly, reef passages attract multispecies fish spawning aggregations (FSAs) (Nemeth 2009; Kobara et al. 2013), which are a critical support for the overall health of reef ecosystems (Erisman et al. 2017). In New Caledonia, a 2010 study based on local knowledge (Ducrocq and Juncker 2010) yielded 274 sites as potential FSAs, most of which were in reef passages or on the fringing reef, and these could be crucial reproduction areas for more than 100 fish species. During the (re)occurrence of these FSAs, fish – including iconic and endangered species (e.g., Aswani and Hamilton 2004; Tupper 2007; Sadovy de Mitcheson 2016; Hughes et al. 2020) – are particularly easy (and enticing) to catch, and thereby extremely vulnerable to over-exploitation (e.g., Hughes et al. 2020; Sadovy de Mitcheson et al. 2020). Hence 26% of surveyed FSAs have shown less abundance in recent years compared to before, and at least two of them have disappeared (Ducrocq and Juncker 2010). Such over-exploitation can eventually alter the natural predator–prey equilibrium (Mourier et al. 2016:2014), given that reef passages also host sharks that regularly feed on these FSAs.

Respective FSA monitoring and survey methods are becoming increasingly efficient (e.g., underwater video technology in New Caledonia, used by South Province) and are regularly generating new knowledge on these biologically rich and complex places. Indeed, this knowledge suggests that multispecies FSA sites should be treated as “ocean bright spots” (Pittman and Heyman 2020), where conservation efforts can replenish exploited stocks and rehabilitate marine ecosystems, and thus accelerate progress towards SDG 14. This recommendation similarly applies to reef passages that are threatened by anthropogenic pressures, including impacts of climate change (aggravating the vulnerability of the reefs; Bell et al. 2011), commercial logging (Hamilton et al. 2019), and boat traffic (Ferrier-Pagès et al. 2021). However, the actual implementation of management measures lags behind (Sadovy de Mitcheson et al. 2020).

In addition to fish and marine species (such as endangered sea turtles) at different life stages, various other living and non-living entities may transit through reef passages: nutrients, sediments, plastics, or chemicals from hinterlands (Graves et al. 2021); as well as boats, fishers, and recreational divers (Doiron and Weissenberger 2014); and sometimes also invisible or supernatural beings (such as spirits of the seas or the souls of the deceased; e.g., Tjibaou 2019).

Relationships between human users and reef passages have received little scientific attention to date, and are mainly presented as exploitation-oriented (e.g., fishers as anthropogenic pressure on these spaces and the resources therein) or conservation-minded (e.g., fishers as members of conservation networks aiming to “[restore] a thriving ocean for the benefit of all”, Pittman and Heyman 2020:6). Yet, our studies in the South Pacific region have led us to consider that relationships between coastal communities and reef passages are not limited to this dichotomy, as also suggested by previous research on islanders’ mental maps (e.g., of the ocean floor around Anuta in the Solomon Islands; Feinberg et al. 2003) as an important part of their local knowledge, and confirmed by other social science literature focused on Oceania.

Since ancient times, reef passages played a role in settlement strategies by facilitating mobility and connectivity, as in the case of Aotearoa New Zealand, where in Hawai’iki Eastern Polynesians actively chose to establish village sites on the coastal flat adjacent to the passage (Kurmann 2018). As reef passages constitute important seascape features for local resource-users, such as fishing grounds or for navigation and transport, it is unsurprising to find detailed local knowledge and terminology referring to reef passages in a number of places (e.g., Hviding 2005). Some reef passages demarcate territories, in particular fishing grounds (Gordon 2010:16), and are also viewed as the visible representation of an important spiritual entity (Gordon 2013), or as guarded by spiritual beings taking the form of, for example, an octopus or a shark (Leblic 2008; Gordon 2010; Aswani 2014). This reflects that seascapes are often constructed and engaged with as spiritscapes (McNiven 2008).

To date, however, very little research in or on Oceania has directly focused on reef passages, how they are perceived, used, governed, managed, and valued by coastal communities, and how the latter relate to the other-than-human elements that dwell in or transit through these spaces. To our knowledge, no systematic literature or policy review on this topic exists, neither for the South Pacific nor any other region in the world. In a context where management and conservation policies and practices are mainly area-based, and reef passages – just like FSA sites – can be identified as priority areas for diverse and healthy oceans, this research gap requires urgent attention.

Through an empirical focus on New Caledonia, this paper presents an exploratory approach to the social-ecological roles of its reef passages. After a brief presentation of our study sites and methods, we explore how different stakeholder groups classify, perceive, and use reef passages. Then, we discuss the implications of these insights into human relationships with reef passages for the management and conservation of these bright spots.

## Study sites and Methods

**Fig. 1:**
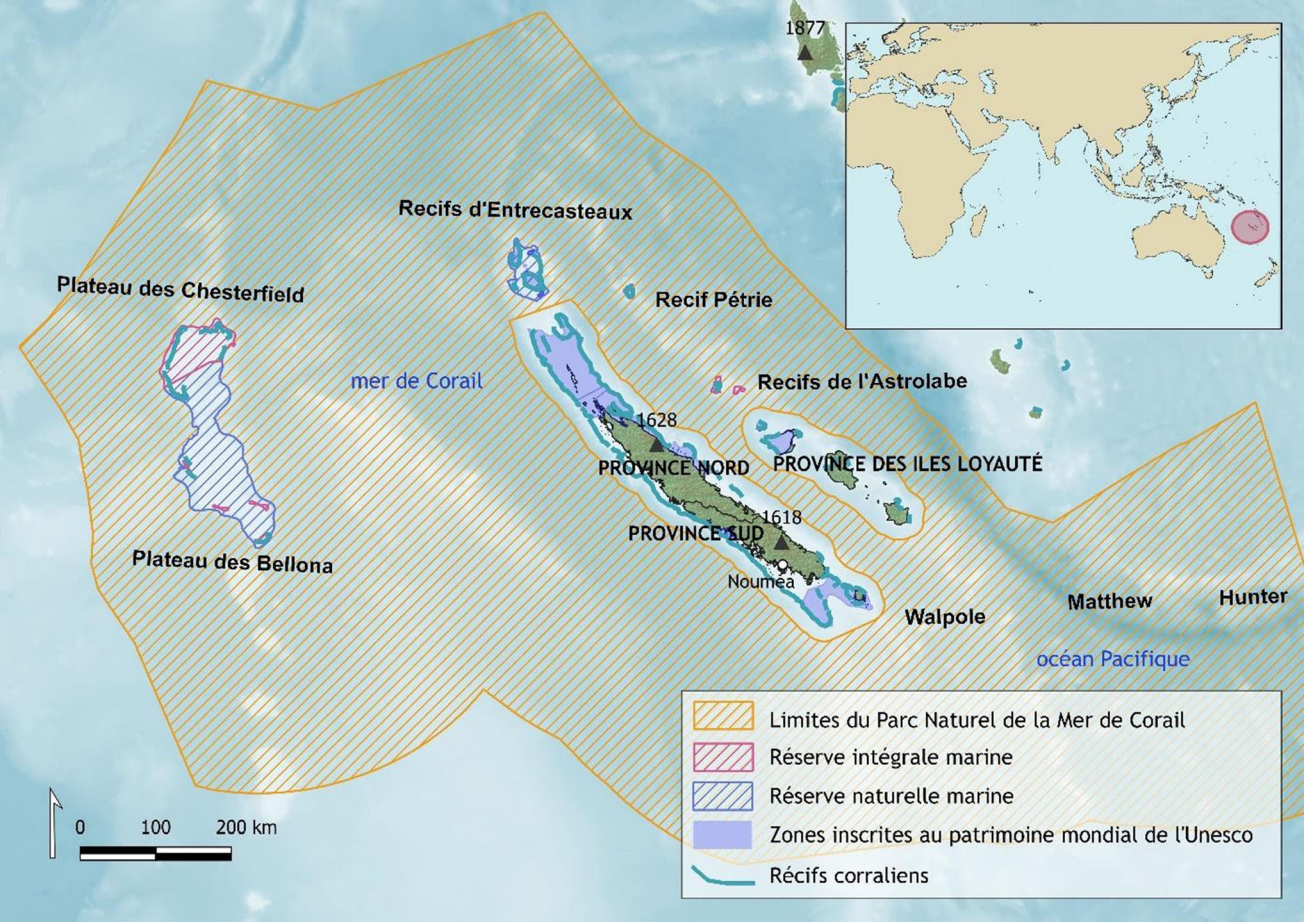
Location of New Caledonia in the South Pacific (small insert), and map of Grande Terre (New Caledonia), showing the diversity of marine protected areas (Parc naturel de la mer de corail, UNESCO sites, and various types of provincial marine protected areas; 'réserve intégrale marine' is the strictest, established to prevent any impact from human activities) (modified from Herrenschmidt et al. 2019)

**Fig. 2:**
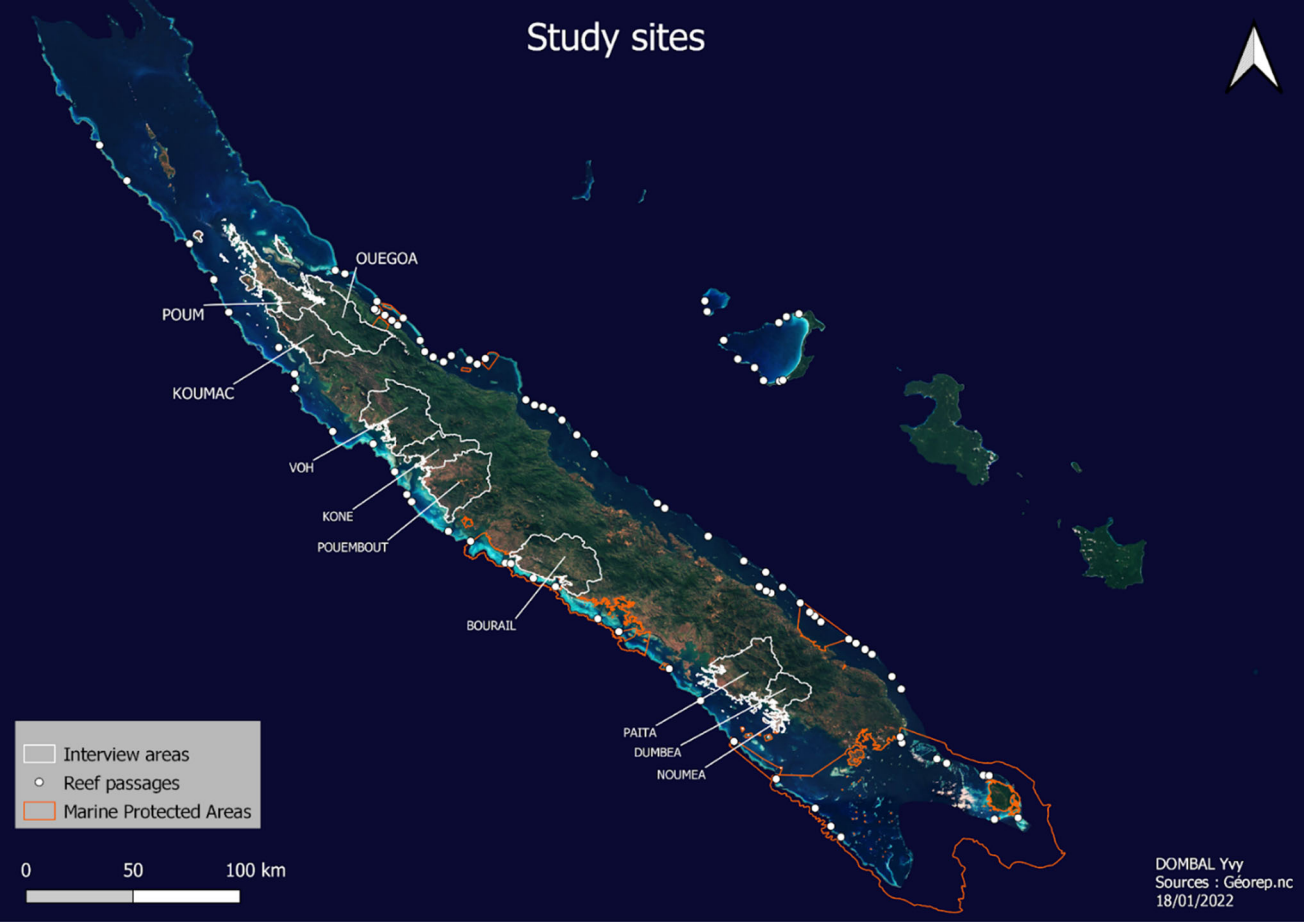
Map of Grande Terre and Ouvéa island (New Caledonia), showing the reef passages and the interview areas (Source: Georep.nc)

New Caledonia is a French overseas territory located in the South-West Pacific region. This archipelago is composed of a main island called ‘Grande Terre’, with a land area of 16 664 km^2^, and several smaller inhabited islands (Maré, Lifou, Ouvéa, Belep archipelago, Île des Pins, Tiga, Île Ouen). The population of New Caledonia is multi-ethnic. About 40% of inhabitants are Kanak, about 25% Europeans, and the rest have various origins, including Oceanian (e.g., Wallis and Futuna, French Polynesia, Vanuatu), Indonesian, and Vietnamese (ISEE 2019). New Caledonia is famous for its almost continuous coral reef around the main island: a ring of submerged reef structures covering 3452.34 km^2^, and ranging in width from 100 to 1000 m (Lasne 2007). Numerous reef passages exist along this barrier reef, some of which are embedded in one of the six marine clusters highlighted by UNESCO as part of the inscription of the New Caledonian lagoons on the World Heritage List in 2008 (see Figs. 1 and 2). Moreover, since 2014, New Caledonia’s entire Exclusive Economic Zone is a nature reserve, the ‘Parc naturel de la mer de corail’. Reef passages are in the jurisdictions of the three provinces of New Caledonia, but can also be bordering the central government’s responsibilities.

Thirty-two semi-structured qualitative interviews were conducted in 2021 on the North and West coast of New Caledonia’s Grande Terre—at Poum, Koumac, Ouegoa, Voh, Koné, Pouembout, Nouméa, Dumbéa, Païta and Bourail (Fig. 2, Tables S1–S3 in Supplementary Information). Locations for interviews were chosen next to passages that were best known to the interviewees. From 93 reef passages (Fig. 2), 40 were directly mentioned by the interviewees (S1).

Interviewees were selected by opportunity- and snow-ball sampling, with interviews being carried out face-to-face (at the most convenient meeting points for the respondents) or, due to the Covid-19 pandemic, via Messenger call. The interviewees were fishers (spearfishing, angling, and trolling; n = 28), scuba divers (n = 4), and surfers (n = 2), with several respondents practising several of these activities.^2^ To progressively refine our understanding of what people know about reef passages and what they do there, our three-level interview guide (S2) was adjusted over the course of the study. This interview guide aimed to collect data on: (1) what reef passages (in general) represent for different stakeholder groups, and what are their social-ecological roles according to the latter; (2) which reef passages are the most frequented within the survey area and what activities are carried out there; and (3) what biophysical conditions and species are observed by the interviewees at these passages.

Informed consent was obtained prior to all interviews, and we followed the ethical guidelines suggested by the International Society of Ethnobiology (ISE 2006). All interviews were conducted in French, lasted on average 1 h, were recorded, and were then transcribed and coded with RQDA (package of R software; Huang 2016). The French transcriptions of all interviews were translated into English by the corresponding author. Their analysis mainly focused on: the local typology and knowledge of reef passages; the different social and ecological roles of reef passages mentioned in the interviews; fishing, scuba diving, and surfing activities carried out at these passages (and tensions between them); and key marine species encountered at these passages.

## Results

### Local typology of reef passages and knowledge of their morphology

Reef passages in New Caledonia are well known to many local residents, whatever community they come from. The respondents mainly talked about the passages closest to their homes, and therefore the ones they have access to most often, but it also happened that they mentioned a strong experience with another passage from times when they were younger. For many, reef passages are the remarkable breaks in the barrier reef that delimit the New Caledonian lagoon, and for some, they also include the openings in the fringing reefs, which were deemed by the interviewees to have the same ecological role but on a smaller scale. These natural passages in the barrier and fringing reefs are differentiated from channels on the reef flats created by humans to facilitate their crossing (Breckwoldt et al. 2022).

Vavouto in Voh (Fig. 2) shows a unique example of a mainly man-made reef passage (Allenbach et al. 2010). The northern nickel company (Koniambo) selected this specific industrial site based on agreements on land tenure, entirely neglecting its access to the sea. To connect this site and its privately owned port with the open sea, a massive passage (4.5 km long, 120 m wide and 12 m deep) was therefore dug by a Spanish enterprise between 2008 and 2010. The Voh reef passage is thus a key part and product of the nickel export systems of New Caledonia.

One interviewee described Voh as ‘lagoon passage’ and commented upon the negative impacts of its creation on pelagic fish:

“*This is a lagoon passage, it is a passage to access, they widened it, they dug it out a bit. This had an effect on all this for a while; it was murky off Voh because they were releasing* [the dug-out material] *quite far away but we noticed that the water was dirty, so it's bound to have an effect on the fish and especially on the pelagics. On the pelagic side of Oundjo when they dug a channel they dug over several kilometres, and the residue there they took and threw it out off the exterior of Voh passage. All the pelagic fish, they did not like the dirty water.*” (Recreational fisher, Voh).

Local interviewees also talked about *‘fausses-passes’*, i.e., ‘false passages’. These, much shallower and therefore not navigable at all tides, can often be seen, for example, as coral ‘collapses’ at the level of the barrier reef, or where so-called spurs and grooves (Rogers 2015) have developed due to interactions of currents and reef growth:

*“So already you will have two terms: you will have the term ‘pass’ and the term ‘false pass’. So here we only have false passes, for example in Bourail. So, it will already be a pass that will be much shallower, more closed, also less wide and where the current will almost always be outgoing.”* (Diving instructor, Bourail).

People who frequently used the different types of passages also had detailed knowledge of their morphology (essential for navigation and for fishing), for instance:

*“If you look closely, there are passes that are very, very narrow, like the Koné pass, it's a pass that's really bad, it's twisted, the big boats will never be able to enter if they don't break or … it's really annoying whereas Voh's pass is quite straight.”* (Recreational fisher, Voh).

Conditions are often more dangerous in these narrow and winded passages compared to the coastal or seaward sides of the fringing and barrier reefs, and require excellent knowledge of (particularly strong) currents, adapted vessels and good weather:

*“La Gazelle* (reef passage) *is a beautiful piece of work! It's the only pass I've crossed, and I was really scared. Because it all depends on how the current arrives […] and it's very impressive when the swell is strong.”* (Recreational fisher, Pouembout).

### Ecological and socio-cultural roles of reef passages as places of value

#### Ecological roles

Reef passages were attributed ecological roles by the respondents, as gathering and transit zones from the ocean to the lagoon (and vice versa) for people, animals, water, and the objects and particles the latter carries.

Reef passages are often reported to be located in front of large rivers, in the prolongation of the river mouths, and most respondents also made a direct link with rivers as the origin of these formations, like an extension of the rivers at sea.

*“The passes are almost in front of the rivers; it is the fresh water that must prevent the coral from growing.”* (Recreational fisher, Pouembout).

As such, reef passages were described as key places for exchange between terrestrial and oceanic waters.

All respondents said that the water carried by the tides through reef passages provides a permanent source of nutrients, hence making these passages attractive places of feeding and reproduction for marine animals, which was often seen as beneficial also for the health and species richness of the nearby adjacent reefs.

*“For me a pass is a break in the coral reef that allows some big fish or rather mammals, maybe whales, dolphins, *etc*., … to be able to enter the lagoon for what they have to do, and yeah I think it allows the exchange of nutrients between the real ocean and then the lagoon…”* (Recreational fisher, Nouméa).

Fishers also regularly pointed out the importance of passages for the spawning aggregations of several fish species, such as king mackerels (*Acanthocybium solandri* and *Scomberomorus commerson*) in Népoui’s passage or speckled blue groupers (*Epinephelus cyanopodus*) in Dumbéa’s passage. The ‘false’ passage of Île Verte was also mentioned as the only known FSA site for napoleon wrasse (*Cheilinus undulatus*).

In addition, reef passages were seen as pollution outlets essential for maintaining clean and healthy lagoons. Rainy events load rivers with sediments and waste of all sorts, which were perceived to be carried towards the open sea by reef passages.

*“We often see plastic buoys, when there’s a lot of rain, everything that’s in the Néra* [river] *flows down there, there’s wood and plastic, everything that people throw into the river goes down to the passes, when we see it we collect it, but I imagine that the rest… “* (Surfer, Bourail).

*“After the big rains, it’s very dirty, especially around the FADs* [Fish Aggregating Devices] *which are generally in front of the passes, you find everything from the woods … It’s dirty.”* (Recreational fisher, Pouembout).

#### Socio-cultural roles

For all interviewees, reef passages were important places for a number of reasons, but first and foremost because they mark the links between Kanak clans, between the visible and invisible worlds, between the living and the dead. Reef passages are pathways to the afterworld for the spirits of the dead, both embodied and accompanied by different animals throughout their journey. In the words of one of our interviewees, these passages are therefore sacred and must not be disturbed but highly respected.

“*For us, for example, in the Aijé-Arö area, the pass in Bourail is the passage of the dead for the entire area. Before, we used to say that when a dead man passed through, you could hear a noise when he took the channel, the passage to get to the bottom of the pass.* […] *They* [the deceased] *change, when you're in the river, we'll say you're an eel;* […] *the eel leaves, it changes, it becomes small, it goes to spawn and then it goes into the channel; by changing, it becomes another totem animal. That's why it's full of fish, why there are turtles; the turtle, the shark are totem animals that are linked to the sea clans.* […] *It's said that the shark also accompanies the dead. Basically, it's as if you go out and you're a remora* [suckerfish, Echeneidae] *and then there's the shark that will accompany you and then you join another world. After all it's a cycle and you'll always come back anyway.”* (Resident of Houaïlou).

Legends were mentioned by some interviewees (e.g., one should not speak badly or say insults whilst crossing a passage to avoid being shipwrecked). A ‘gesture’ towards reef passages, i.e., an offering (e.g., tobacco, fabric, money, or food) that one makes to the sea, sometimes accompanied by a few words, was said to placate the spirits therein, and to preserve those who go there.

*“The passes are sacred; it is the passage to the world of the dead, the spirits of the dead are there, we avoid disturbing them, we have a fear when we fish there, we don't go alone, always a small gesture to ask the old people for forgiveness and to look after us.* (Professional fisher, Koné).

This gesture was also a way of remaining humble towards the sea, yet not anymore observed by all interviewees.

*“I don't make a gesture but my grandparents did at the time, it's not everywhere, it's just at the Amos pass, we have a false pass on the side, we call it taboo, so as it's taboo, you have to make a gesture when you leave and when you arrive, but I don't do it, I used to do it with my grandparents when I was a child.”* (Professional fisher, Puebo).

For important customary/community events (such as weddings in Poum), residents were allowed to access the passage to fish for specific species such as napoleon wrasse, to be consumed during the feast, even if this passage was considered sacred or ‘taboo’ and fishing was forbidden there for the rest of the year. Hence, in a taboo zone the activities are not entirely and always forbidden, but they have to be carried out with respect and sometimes only after getting authorisation by the clan elders.

Finally, people talked of reef passages as ‘openings to the world’. They have indeed, for a long time, allowed trade and ensured the transport of people (and thus of news) as well as of products and raw materials from the outside to the ‘inside’ of the island, and vice versa. Many shipwrecks have occurred near these places, and some passages are therefore named after famous ships or navigators of the time.

### Interviewees’ activities in passages

The interviews confirmed that reef passages were used mainly by: a) fishers (professional, recreational, and subsistence ones), b) scuba divers (by boat) and, to a lesser extent, c) surfers.^3^

#### Fishing

The interviewees reckoned that reef passages are suitable for different types of fishing (spearfishing, trolling, angling). However, deemed dangerous and sometimes very remote from the shore, reef passages were not necessarily seen as the best place for all these fishing techniques. Besides for fishing within these passages, fishers also used them for transit to get to the FADs in the open sea. Several fishers mentioned making a gesture (see above) at the passage to ensure a smooth navigation and a good catch.

Spearfishing with underwater guns is a very popular form of selective fishing in New Caledonia. In general, this activity is practised by a minimum of three people, two in the water and one on the boat to ensure the safety of divers. Spearfishers like to fish at passages because there are always fish readily available there. However, fishing in a passage requires good physical ability and favourable weather conditions. Interviewed spearfishers had an excellent knowledge of their fishing grounds, knowing the distribution of consumable species and the (geo)morphology of the area.

*“…The right side of Uitoé* [passage] *when you look out to sea is big steps, things where the groupers can rest quietly, they have space, they have each other you see, so it's a grouper spot. After that, you have spots on the left of Uitoé with more coral, as there are coral descents with a little bit of caves, you can find big groupers hiding, like big mother groupers, all the species of groupers … the big blue groupers in the passes, the big parrotfish on the left; you will find some on the right but less … as it will be more of a drop off, and straight into the pass, if you are lucky, you will come across some jacks *(Carangidae)* that sometimes come out to see what you are doing, a kingfish if you are lucky…”* (Recreational fisher, Nouméa).

Trolling is a fishing practice that consists of towing a line with a bait, lure or live fish behind a moving boat at a slow or medium speed. In New Caledonia, fishers who practice this type of fishing usually have a boat with an engine of long-distance capacity, adapted to big game fishing.

*“At the central pass (Poindimié), I look for kingfish, rainbow tuna, and coral grouper. And I often catch bonito jacks, mackerel, and barracudas. I go to the pass to target these big fish that are not found in the lagoon. […] It's a channel between the coral reef, with sandy bottoms from 20 to 50 m depth approximately, with some shallow waters with pebble potatoes *[little coral heads]* down to 3–4 m, and on the exit of the channel, it drops quite quickly to a hundred metres deep. With a map of the seabed, you can see these shallows that often have higher fish concentrations, you pass close to these shallows for trolling, you jig*^4^* on the edge of these shallows.”* (Professional fisher, Koné).

Some anglers (from boat, no live baits) go specifically to reef passages to target certain species (e.g., kingfish and parrotfish). Some fishers in Bourail mentioned that they would not usually go fishing in the ‘false’ passage of Poé but go there sometimes to get shelter from wind and waves.

*“There are much easier areas* [to catch fish]*. But the good thing about this area is that it's protected from the swell and the wind, so it's quite useful when the conditions are not great for several days in a row*." (Professional fisher, Bourail).

#### Scuba diving

Scuba diving is a widely practised activity—by both men and women—in New Caledonia, where there are about 20 diving centres visited by New Caledonians as well as tourists. Divers often look for places where they can find the highest biodiversity and therefore many different animals to observe, without having to dive very deep or travel very far. Reef passages are therefore often favourite dive spots, especially when they attract iconic species (e.g., sharks, napoleon wrasse, or sea turtles), and host FSAs, for instance of groupers:

*“At the big Kélé bend we have a school of between 20 and 30 female grey sharks for the most part. *[…]* We will soon arrive at the end of the year, so the grouper breeding season is mainly in December, and we will have a gathering of hundreds of groupers, so it will be really beautiful, we will have a lot of jacks, barracudas …”* (Dive instructor, Bourail).

#### Surfing

In New Caledonia, surfing is not as widely practised as fishing or scuba diving, which are very popular regardless of location, community affiliation, and age. According to the Caledonian surfing league, which had 400 members in 2016, there were half as many women as men. Seven surfing clubs^5^ are gathered in the Caledonian surfing league and attached to the French surfing federation. Surfing encompasses all practices of gliding in the waves, without restriction of posture (standing, lying, kneeling) or board type (incl. kite surfing). New Caledonian surfers tend to use long boards that are suitable for both small and large waves. Only few surf spots are accessible from the coast, and the main ones are in proximity (left or right) of passages, providing surfers with a good knowledge of these places and their current conditions. For example, at Ouano passage, Dumbéa passage, Ténia passage, Mato passage, Gouaro passage, or Bourail passage, there are well-known surf spots. Bourail passage is one of the most interesting ones according to the interviewed surfers, because there it is possible to surf from the beach to the left side of Île Verte in the middle of the bay.

*“The swell when it comes in, it rushes into this channel * (Bourail passage) *too, so the swell from the open sea is formed into this channel. When the wave arrives, it meets the reef on the right, it breaks and as it breaks, it rolls off the reef; as it rolls off, it gives a break zone which allows you to surf, otherwise it would just be a wave that crashes. The waves depend on the swell, the swell has to be from the west, and from the size of the swell obviously, up to 0.1 m to 1 m;* […] *the more it is west, the more it comes in and it comes in at the beach too.”* (Surfer, Bourail).

One spot at Gouaro was mentioned as the least dangerous, narrowest spot, which makes it better for beginners, and which is always surfable, depending on the tides. Another well-known spot is in proximity to the Ouano passage, 10 km away from the shore of la Foa where competitions are usually held.

*“The Ouano pass is super beautiful, it can go very far, you can have 300–400 m of zone from the take off*^6^*to the end of the wave, it is world famous even, there are foreigners who come to surf here, it is a super quality wave!”* (Fisher and former surfer, Dumbéa).

#### Tensions between fishing and other activities

Although the surf spots are quite explicit, they are sometimes frequented by spearfishers, and the proximity of people ‘hunting’ underwater can disturb some surfers, particularly regarding the risk of shark attacks.

*"In Dumbéa, for example, it's well known that you have the false pass 1 and the false pass 2 *[…]* To spot it, you see a lot of surfers who are often on it, but then there are also fishermen. So, there too, there are conflicts, especially when there were shark attacks last year, and I know that the surfers who were there on the false pass spots were fighting against the fishermen who were fishing in the false passes. Because they said that it would attract sharks and the others said well, we don't care, we've always fished there, it's not going to change now. So, I know that it was a bit tense for a while around the false passes.”* (Fisher and former surfer, Dumbéa).

Similarly, scuba diving activities can occur in parallel with the fishers’ activities, which can also lead to tensions.

*“On the other hand, I've seen fishermen in the passes, for example, at the ‘shark faille’, where you're scuba diving next to them and it's very dangerous, it's forbidden to fish, you're in the reserve, and the guys are with their spearguns, waiting in the current and shooting. So I went to see them because they were diving, it's dangerous scuba diving, at the ‘shark faille’ there's not good visibility so you don't know with their spearguns if they recognize you're diving; so no, it's a potential risk of accident, of food stimulation for, well there are greys* [grey sharks] *that turn into the pass, and well yeah, afterwards you go to see them, and they say: here, I'm at home and you're not, and that's it … “* (Diver, Bourail).

These two quotes point towards tensions, potentially due to: a) perceptions by some surfers and scuba divers that fishers’ activities present a danger for other reef passage users, or at least attract dangers (e.g., risk of shark attack); and b) fishers having a particularly strong attachment to the passages and a consideration of themselves as more legitimate users than surfers and scuba divers. These above quotes also hint towards a possible mismatch between the statements (mainly of surfers and scuba divers) that reef passages need better protection and fishers’ perceptions that there are already enough protected areas (especially when these are next to their jetties so they have to cross all the reserves to get to their fishing area). For example, in the case of the ‘false pass’ of Île Verte near Poé, a protection of the passage would only be accepted in exchange for the removal of the (touristic) Poé protected area.

"*There are already too many reserves at Bourail, if you listen to them* [non-fishers] *there's never enough. There is no need for the ‘false pass’ reserve. The interest of a reserve is the protection of several habitats, there is no proven ecological interest here, the spawning area, the last report I read it was not more a spawning area than other places. The interest of the current reserves, close to inhabited areas, is protection from direct over-exploitation but the downside is we must go further. We forget that people don't have the money for petrol, where will they go next? In my opinion, the ‘false pass’ reserve was originally designed to favour the diving clubs who were fed up with being next to the hooks. But anyway the ‘false pass’ is too deep for us hunter divers and it's not practical for the seine.”* (Professional fisher, Bourail).

#### Key species in reef passages

All interviewees highlighted the species richness of reef passages, including that of large predators. Sharks were mentioned exceptionally often, most frequently grey reef (*Carcharhinus amblyrhynchos*), sharpnose (or small blacktip, *Carcharhinus limbatus*), hammerhead (*Sphyrna mokarran*), bull (*Carcharhinus leucas*), and blacktip sharks (*Carcharhinus melanopterus*). The passages were indeed mentioned as those places where chances are highest to encounter big sharks and sometimes several dozen individuals.

"*In the passes, we often observe the grey shark and then the others with the pointed nose *[Carcharhinus limbatus]* that swim a little faster, and then hammerhead sharks. In the years I've been diving, I've only seen one tiger shark underwater, and there aren't many. Hammerhead sharks more often, and then the others, the grey ones, and then the pointy-nosed ones, always in the passes, that always eat the fish, it depends on the periods, sometimes there are a lot of them* [e.g., for feeding or resting in the current]*, sometimes there are not many*.“ (Recreational fisher, Bourail).

One of the testimonies collected from a Kanak fisher shows particularly well how important the shark is from both an ecological and a socio-cultural point of view:

*“The shark has always existed, it's part of our totems, if it's there it's because it has a job to do, and especially in the zone of the passes, you see we say that it's there in the path of the dead because it accompanies, but because there's also food there, so unconsciously we'll say well, it's the path of the dead, but when you look at the thing, it's full of nutrients inside *[…]* It's a cycle that already exists, but yet the old guys of us* [elders and ancestors]*, they didn't do science.”* (Fisher, Koné).

Kanak fishers recognized the local ecological knowledge of sharks built by their ancestors and noticed the concordance of this knowledge with the scientific knowledge of which they became aware only later.

## Discussion

The knowledge of New Caledonian reef passages held by the coastal stakeholders interviewed in this study was substantial and multifaceted, in terms of the different names that existed for the different types of passages, their morphological features, currents, and fauna. This mirrors results of in-depth studies in other parts of the South Pacific region, for example the Solomon Islands (Feinberg et al. 2003; Hviding 2005; Aswani 2014). This exploratory study on New Caledonian reef passages also provides a first glimpse on local perceptions of their essential ecological and socio-cultural roles—for humans (in the Kanak cosmology but also in terms of important fisheries, transport, and biodiversity protection), for fish (large and small), and for the surrounding reef environment.

This knowledge and awareness were for many interviewees a reason to acknowledge the need to protect these ecological and cultural keystone places,^7^ at and from which ecological and cultural keystone species^8^ live. These places and species are relevant for biodiversity protection and ecosystem health, as well as the intertwined human health and well-being (e.g., Betley et al. 2021). Many interviewees were thus strongly motivated towards conservation of reef passages and respective marine protection rules, especially to preserve these places for the species gathering there (to be harvested, admired, etc.). At the same time, as pointed out by some interviewees, large parts of New Caledonia’s coast are already covered by protected areas. Several passages are even located within different types of protection zones—in the Natural Park of the Coral Sea itself, in the UNESCO western coastal area (Zone Côtière Ouest), in a so-called ‘sustainable management area’ (Aire de Gestion Durable; in Puébo), or in a ‘wilderness reserve’ (Zone Sauvage; in Touho). Other passages are partially managed under customary taboos (e.g., Poum passage), and Dumbéa passage is a rotating reserve, particularly for the FSA of blue groupers. Some users (especially fishers) therefore question, or even oppose, the implementation of additional protection zones or tools.

Besides such important aggregations of finfish species, sharks came up frequently in the interviews, as ecological and cultural keystone species for all the mentioned reef passages, also indicating the large amounts of sharks that can regularly be found at (and close to) these places. This abundance of sharks, together with the protected area discussions, caused some tensions—between scuba divers and fishers, as well as between surfers and fishers (especially spearfishers who were said to attract additional sharks due to wounded target fish). A holistic lens is needed for timely solutions to these tensions, including considerations that integrate the recent ‘shark crisis’ following lethal shark attacks on fishers and surfers in New Caledonian waters.^9^ It could be relevant to understand how people who are both scuba divers and fishers, or both surfers and fishers, deal with or approach these tensions, as a first step to integrate and address the latter in management and conservation discourse and practice. A shared recognition that reef passages can only be protected with joint efforts by all user groups needs to be achieved (e.g., via activities emphasizing common ground rather than differences) and could eventually help navigate these tensions, considering that friction per se can also stir new developments.

To protect New Caledonia’s reef passages as keystone places therefore seems to be not only a question of simply increasing the number of protected areas (and closed-off space), but rather to also change the character and quality of the existing protection, thereby increasing the level of respect and acceptance of protection criteria. Accounting for local priorities and forms of usage of marine areas such as reef passages (e.g., with temporary use options), including immaterial values people assign to these places, can help to engage local people, acknowledge their connection to marine places, and ultimately contribute to marine conservation (Feinberg et al. 2003; Ferse et al. 2010; Sterling et al. 2020). Customary tenure and taboos cover many coastal and marine areas not covered by formal conservation arrangements, and whilst they can be differentiated from marine protected areas in that they frequently do not have conservation as their primary goal (Foale et al. 2011), they can still yield relevant conservation results. These so-called Other Effective area-based Conservation Measures (OECMs) are increasingly recognized for their potential to achieve equitable conservation and livelihood outcomes (Gurney et al. 2021), with countries like Indonesia currently working on their formal legal recognition (Estradivari et al. 2022). Such OECMs would fit particularly well to reef passages in New Caledonia, where some are already covered by existing customary rules, e.g., at Poum. These may thus already qualify as OECMs, where all marine species (predators and prey), and the living and dead humans, can find their place.

In a ‘One Health’ approach, deemed as critical for achieving the UN 2030 Agenda for Sustainable Development and the related SDGs (FAO 2011), healthy reef passages, and healthy and well-managed reef-related species, can be seen as vital resources for healthy people and their well-being (e.g., Sterling et al. 2020; Betley et al. 2021). Well-being would then include the ability to care for both human and other-than-human beings, as well as to take care of the deceased in a culturally appropriate way, i.e., being accompanied by a shark to the open sea and afterworld. In this perspective, the presence and powers of the spirits are seen just as important as the ecological roles of reef passages (e.g., to be able to see or catch a big grouper). In addition, the values of reef passages can be influenced by different myths, stories and legends, ancestral and modern, carried by Kanak people, as well as by fishers, scuba divers and surfers of other origins.

The roots of the Kanak world and all its elements are indeed based on myths. In these myths, all human and other-than-human elements from the sea and land areas are interconnected (Dégremont and Sabinot 2020). The origin of many essential elements of Kanak culture is found in the sea, such as the remedies to heal oneself or the Kanak currency (Leblic 2008).^10^ In the Kanak worldview(s), there is no discontinuity between ‘culture’ and ‘nature’, or between human and other-than-human entities (hence, the relevance of the concepts of ecological and cultural keystone places and species). The living and the dead are strongly linked (e.g., via the rivers), as are the visible and invisible worlds. In this representation of the world, the souls of deceased elders are omnipresent in the environment and in the lives of Kanak people, particularly at each important stage of their lives, such as births, marriages, and deaths. Deaths in Kanak society are events marked by ceremonies that allow the soul to be detached from the body of the deceased, to then take the path of the dead. In its journey, the spirit of the dead leaves the interior of the land, passes through the rivers, leaves the lagoon through reef passages, and finally reaches the sea where it joins the world of the dead (Ogier-Guindo 2011; Tjibaou 2019).

Compared to these ancestral myths that connect people to land and sea, modern myths might be ranked as less important culturally, yet they can also play a significant role for the engagement of local people in activities (e.g., by bringing tourists to the reef passages). Modern myths may include those conveyed by scuba divers (told as stories on the dive boats taking them to and from the passages and back on land with friends and family), often surrounding type, size, and behaviour of sharks and other remarkable keystone species, as well as currents, visibility, and encountered intricacies of the reef morphology. Tourist fishers may contribute their stories of the exhilarating battle with the fish, the profusion of various fish species, or the intricacies of navigating these dangerous areas; and surfers may contribute their own stories on these aspects, as well as on specific wave conditions in the reef passages. Accounting for the importance of these different local myths, knowledges, and philosophies, it seems important to contrast them with each other, in order to a) further illustrate the complexity of the afore-mentioned tensions, yet b) create a shared space (i.e. with the reef passage as boundary object) to jointly tackle the management and protection of reef passages and their local use(r)s.

Therefore, whilst we have identified a gap between how scientific references to reef passages are scarce and scattered, and how reef passages appear of multifold ecological and cultural importance in New Caledonia (see S3), this study is a first step highlighting the necessity of, and providing the foundation for, extended research on reef passages also in other countries across the tropical belt. Our study adds knowledge on both the ecological and socio-cultural significance of these keystone places, which should be more explicitly considered under existing and future management and conservation measures. Accounting for the multiple uses, knowledges, and values of reef passages indeed has the potential to increase the social-ecological efficacy and meaningfulness of these measures, including formal regulations.

## Conclusion

In New Caledonia, reef passages are conceived as areas where rituals and taboos are performed; areas of transit to the outside (including for the spirits of the deceased as well as emblematic and totemic species); areas through which everything from the outside enters the islands and affects societies in either a ‘good’ or ‘bad’ way (e.g., boats, pollution).

Inhabitants frequently use reef passages for fishing, scuba diving and surfing. Here, high-value pelagic fish can be caught near-shore, daily, without having to venture out into the open sea. Scuba divers can encounter many animals (including sharks) without having to dive very deep or travel very far, whilst surfers can find good spots, adapted to their various preferences and skills.

The study has shown that, in New Caledonia, reef passages are considered ‘communication zones’ between coastal and oceanic spaces and species, and have significant yet un(der)explored ecological and socio-cultural roles. In our view, inter- and transdisciplinary research on reef passages is all the timelier as many people depend on them for food security and livelihoods; at the same time, they are increasingly threatened by climate change, pollution, and overexploitation. Further research on reef passages will thus contribute to global conservation agendas, aiming at interdisciplinary and inclusive procedures (e.g., concerning local knowledge) surrounding SDG 14 (Life below water), and specifically the target to protect 30% of the Earth’s surface by 2030 (and resistances to it). For the ‘Large Ocean Island States’ of the South Pacific, these 30% will invariably cover to a large extent seascapes, potentially including reef passages.

## Supplementary Information

Below is the link to the electronic supplementary material.

Supplementary file1 (DOCX 41 KB)

## Footnotes

1 https://www.cbd.int/coral-reefs/commitments

2 Various cultural communities composed our sampling, with an important proportion of Kanak and European people born in New Caledonia; a future study will include a focus on gender, cultural, ethnic and professional backgrounds.

3 Gender did not (yet) play a role in our interviewee selection and analysis, as the user activities as such were in focus, none of which were reserved to men.

4 Jigs are lures specially designed for fishing predatory fish.

5 Due to the "shark crisis" (six attacks by sharks in 2020 and three in 2021), the member numbers have dropped, and competitions have been frozen for two years.

6 To stand on the board just when starting to catch the wave.

7 Elaborating on the work by Cuerrier and colleagues (2015; Lepofsky et al. 2017), who developed the term and concept of Cultural Keystone Places (CKPs) for terrestrial locations “with high cultural salience for one or more groups of people”, and which play, or have played in the past, “an exceptional role in a people's cultural identity, as reflected in their day to day living, food production and other resource-based activities, land and resource management, language, stories, history, and social and ceremonial practices” (Cuerrier et al. 2015:431).

8 Building on Garibaldi and Turner’s (2004) definition of Cultural Keystone Species (CKS)—“culturally salient species that shape in a major way the cultural identity of a people” (Garibaldi and Turner 2004:4; see Butler et al. 2012 for a marine example), and on Cristancho and Vining’s (2004) definition of Culturally Defined Keystone Species—"those plant and animal species whose existence and symbolic value are essential to the stability of a culture over time" (Cristancho and Vining 2004).

9 https://la1ere.francetvinfo.fr/nouvellecaledonie/province-sud/noumea-0/le-plan-d-action-contre-le-risque-requin-de-la-province-sud-a-ete-devoile-1196356.html

10 The Woman, the Mother, is also clearly linked to water, to the Ocean (Leblic 2008).
