# Supplementary material for: A social-ecological engagement with reef passages in New Caledonia: Connectors between coastal and oceanic spaces and species
Source: Ambio. 2022 Aug 18;51(12):2401–13. doi: 10.1007/s13280-022-01762-8 (PMC9386666; doi:10.1007/s13280-022-01762-8)
Supplement: Supplementary file 1 — Supplementary file1 (DOCX 41 KB) [file 13280_2022_1762_MOESM1_ESM.docx]

***Ambio***

Electronic Supplementary Material

*This supplementary material has not been peer reviewed.*

Title: **A social-ecological engagement with reef passages in New Caledonia: Connectors between coastal and oceanic spaces and species**

Authors: Annette Breckwoldt, Yvy Dombal, Catherine Sabinot, Gilbert David, Léa Riera, Sebastian Ferse, Elodie Fache

S1 - Supplementary information on the conducted interviews

Table S1: Details of 32 interviews and interviewees

| Localisation | Age | Activities type | Interviews type | Misc. |
| --- | --- | --- | --- | --- |
| Bourail | 53 | recreational fisher | face to face |  |
| Bourail | 23 | recreational fisher | face to face |  |
| Bourail | 32 | scuba diver | face to face |  |
| Bourail | 40 | scuba diver | face to face |  |
| Bourail | 44 | surfer | face to face |  |
| Dumbéa | 33 | recreational fisher | messenger call |  |
| Dumbéa | 49 | recreational fisher | messenger call |  |
| Koné | 27 | recrational fisher | face to face |  |
| Koné | 42 | professional fisher | face to face |  |
| Koné | 41 | recreational fisher | face to face |  |
| Koné | 45 | professional fisher | messenger call |  |
| Koné | 43 | elderly professional fisher | face to face | shark oriented |
| Koumac | 27 | recreational fisher | face to face | shark oriented |
| Koumac | 49 | recreational fisher | face to face | shark oriented |
| Nouméa | 24 | recrational fisher | messenger call |  |
| Nouméa | 26 | recrational fisher | messenger call |  |
| Nouméa | 36 | recrational fisher | messenger call |  |
| Nouméa | 26 | recrational fisher, scuba diver | messenger call |  |
| Nouméa | 56 | recreational fisher | messenger call |  |
| Ouegoa | 62 | scuba diver | face to face | shark oriented |
| Ouegoa | 58 | elderly professional fisher | face to face | shark oriented |
| Ouegoa | 44 | recreational fisher | face to face | shark oriented |
| Ouegoa | 28 | subsistence fisher | face to face | shark oriented |
| Païta | 26 | recreational fisher, surfer | face to face |  |
| Poindimié | 38 | recreational fisher | messenger call |  |
| Pouembout | 31 | recrational fisher | face to face |  |
| Pouembout | 30 | recreational fisher | face to face |  |
| Pouembout | 28 | recreational fisher | face to face | shark oriented |
| Poum | 65 | subsistence fisher | face to face | shark oriented |
| Poum | 34 | recreational fisher | face to face | shark oriented |
| Poum | 56 | recreational fisher | face to face | shark oriented |
| Voh | 46 | professional fisher | face to face | shark oriented |

Table S2: Municipalities where interviews were conducted

| Municipalities | Provinces | Distance from Nouméa | NB of inhabitants | NB of reef passages |
| --- | --- | --- | --- | --- |
| Nouméa | South | / | 94.285 | 0 |
| Païta | South | 30km | 24.563 | 1 |
| Dumbéa | South | 17km | 35.873 | 1 |
| Bourail | South | 175 km | 5.531 | 4 |
| Pouembout | North | 272km | 2.752 | 2 |
| Koné | North | 280 km | 8.144 | 1 |
| Voh | North | 314 km | 2.856 | 1 |
| Koumac | North | 386 km | 3.981 | 3 |
| Poum | North | 445 km | 1.435 | 2 |
| Ouégoa | North | 430 km | 2.118 | 2 |
| Poindimié | North | 316 km | 5.006 | 4 |
|  |  | source DITTT | source ISEE 2019 | source Géorep |

Table S3: Overview of 93 reef passages in New Caledonia (Grande Terre and Ouvéa) – 40 cited (yellow) by the interviewees

S2 - Interview guide

| Projet SOCPacific | | |
| --- | --- | --- |
| ►Contextualiser la pêche dans la région du Pacifique Sud en explorant le vaste réseau de connexions socioculturelles, géopolitiques et politiques dans lequel s'inscrivent les pratiques de pêche. | Ce projet se déroule dans trois région : Nouvelle-Calédonie, Vanuatu et Fidji. (2018-2021) | Trois domaines thématiques : **1) Les valeurs sociales des lieux et des ressources en relation avec les pêches et la pêche côtière** 2) L'imbrication des questions relatives à la pêche et à la conservation, au sein des protégées et gérées 3) L'inclusion des pêcheries dans les cadres émergents de planification de l'espace marin. |
| Métadonnées : Nom, prénom, âge, sexe, emplois, lieu de vie, appartenance éthnique. | | |
| **Guide d'entretien** | | |
| Thématiques | Questions générales | Questions à poser |
| Passes en générale | Si je vous dis passe récifal, à quoi pensez-vous ? |  |
| Définition de passe récifale | Conceptualisation d'une passe | Pouvez-vous me définir ce qu’est une passe pour vous ?  Que représente-elle pour vous ? dans votre culture ? A quoi servent les passes ? Quels rôles ont-elles pour les Hommes, pour l’environnement ? Pouvez-vous lister les animaux/plantes/autres êtres 1) qui vivent dans les passes ; 2) qui transitent par les passes ; 3) qui ont besoin des passes ? Y a t-il souvent des navires qui transitent par les passes? Quel genre de navire ? Avez-vous observé d’autres objets ? Plastique, tronc d’arbres, pierre ponce, algues ….  Que se passerait-il s’il n’y avait plus ces connexions lagons/haute mer ? Que permettent-elles ?  Quel est la différence entre les passes du récif barrière et les passes du récif frangeant ? Fonctionnent-elles de la même façon ? Y retrouve-t-on les mêmes conditions ? Les espèces qui y circulent sont-elles les mêmes ?  Y a-t-il des passes protégées ? lesquelles ? Pourquoi ? Comment ? (Aire marine protégée ou autre) Y a-t-il des passes tabous ? Y a-t-il des choses à faire ou des choses interdites avant d’aller là bas ? Une fois arrivé à la passe, y a til des choses à faire (un tabac, un rituel ?) et des choses à ne pas faire ? Pouvez-vous me citer les passes que vous connaissez ? |
| Toponymie | Origine des noms ? | Y a-t-il une histoire liée à ces noms ? d’animaux, des histoires, des spécificités géographiques, topographiques, des courants A est elle un autre nom en la langue ou autre ? |
| Passe utilisées/fréquentées | Quelles passes fréquentés vous ? |  |
| Activités | Quelles activités y pratiquez-vous ? |  |
|  | Pêche | Est-ce que vous péchez dans les passes ? dans la passe même ou à proximité ?  Lesquelles ? Vous y allez à quelle fréquence ? Avec qui ? Comment (bateau ou non par exemple) ? Quelles techniques de pêche utilisez-vous ? Pourquoi vous péchez dans là-bas ?  Vous y péchez quoi ? Il y en a beaucoup de ces espèces, en quelle quantité à peu près ? |
|  | Plongeurs bouteille et autres activités nautiques | Quel est l’intérêt pour un plongeur/surfeurs, … etc. de plonger dans une passe ?  Quelles sont vos passes (spot) préférés ? Pourquoi ?  Vous y allez souvent ? Avec qui ? Comment ? |
|  | Autres | Pourquoi utiliser vous cette passe ? Pour déplacements ? D'ou venez vous et vers ou allez vous ? L'emprunter vous souvent ? |
| Focus sur une passe ou un ensemble de passes spécifiques | Caractériser une/des passe(s) précisement |  |
| Biodiversité et abondance | Qui vit dans la passe ? | Quelles espèces y observez-vous ? (Lister de façon exaustive) Vivent-elles dedans, ou sont-elles juste de passage ?  En quelles abondances ? Quelques individus, 50, 100, +100 Quelle taille ont les individus ?  Quand est ce qu’on les voit le plus ?  Est-ce une zone de frai d’espèces de poissons ? Lesquelles ?  Y a-t-il beaucoup de coraux sur les pentes ? Pouvez-vous me décrire les espèces et/ou citer leurs genres ?  Et les alentours ? Il y a-t-il beaucoup de poissons aussi ? est-ce les mêmes espèces ? le récif est-il en bonne santé ? |
| Conditions | Quelles sont les conditions dans ces passes ? | Est-elle proche de la cote ? Quelle est sa distance à la côte ?  Qui y a-t-il a proximité ? est ce que ça a une influence sur les passes ? (rivières) Est-ce que cette passe est toujours navigable ? (En fonction des marées …) Est-elle facilement accessible? Agitation de l’eau? plus/moins agités que dans le lagon et/ou en haute mer Comment est le courant ? fort moyen faible, constant, périodique ? Dans quel sens est-il ? Change-t-il souvent de sens ?  Turbidité ? Il y a une bonne visibilité ?  Il y a combien de profondeur ?  Il y a quoi comme fonds ? Sableux, rocheux, …  Avez-vous observé une différence avec l’effet de la Nina sur les passes ? |
| Changement | Evolution ? | La passe a-t-elle changé depuis que vous la connaissez ? A quoi est-ce dû (évenement climatique intense ) ? Qu'est ce qui a changé ?  Ont-elles subi des aménagements ? élargissement, approfondissement, voie navigable. Si oui quand ? Pourquoi ? de quelle manière ? par qui ? |

S3 - Box: Future research questions

1. What other features make reef passages ecologically and culturally significant places, and can their economic significance be quantified?
2. What role does the impact of boat traffic/transport play - for the activity itself (fishing, diving, surfing) or simply for transfer to reach shore, which can have a considerable impact itself (e.g., due to noise, or collision with marine life)?
3. How can the sources and impacts of pollution best be understood and quantified, and this knowledge used to influence the health of the reefs?
4. How can the local typology and knowledge of the morphology of reef passages be refined by research using on-site ground-truthing coupled with satellite-based imagery (Breckwoldt et al. in review)?
5. How can the differences of people’s knowledge (depending on the activities they carry out in the reef passages) be characterised?
6. How could the policy landscape be equipped, at both the national and regional levels, to secure the multiple uses and values of reef passages, while mitigating existing tensions between users?
7. How to recognize and combine these various aspects surrounding reef passages for conservation, management, and marine governance between provincial, governmental and national responsibilities? Where is more protection needed and meaningful?
